# Therapeutic Stomatocytes with Aggregation Induced Emission for Intracellular Delivery

**DOI:** 10.3390/pharmaceutics13111833

**Published:** 2021-11-02

**Authors:** Jingxin Shao, Shoupeng Cao, Hanglong Wu, Loai K. E. A. Abdelmohsen, Jan C. M. van Hest

**Affiliations:** Bio-Organic Chemistry, Institute for Complex Molecular Systems, Eindhoven University of Technology, P.O. Box 513, 5600 MB Eindhoven, The Netherlands; j.shao@tue.nl (J.S.); caos@mpip-mainz.mpg.de (S.C.); h.wu@tue.nl (H.W.)

**Keywords:** biodegradable stomatocytes, aggregation-induced emission, enzyme cross-linking, autonomous motion, intracellular delivery, anticancer therapy

## Abstract

Bowl-shaped biodegradable polymersomes, or stomatocytes, have much potential as drug delivery systems, due to their intriguing properties, such as controllable size, programmable morphology, and versatile cargo encapsulation capability. In this contribution, we developed well-defined therapeutically active stomatocytes with aggregation-induced emission (AIE) features by self-assembly of biodegradable amphiphilic block copolymers, comprising poly(ethylene glycol) (PEG) and AIEgenic poly(trimethylene carbonate) (PTMC) moieties. The presence of the AIEgens endowed the as-prepared stomatocytes with intrinsic fluorescence, which was employed for imaging of cellular uptake of the particles. It simultaneously enabled the photo-mediated generation of reactive oxygen species (ROS) for photodynamic therapy. The potential of the therapeutic stomatocytes as cargo carriers was demonstrated by loading enzymes (catalase and glucose oxidase) in the nanocavity, followed by a cross-linking reaction to achieve stable encapsulation. This provided the particles with a robust motile function, which further strengthened their therapeutic effect. With these unique features, enzyme-loaded AIEgenic stomatocytes are an attractive platform to be exploited in the field of nanomedicine.

## 1. Introduction

Supramolecular polymeric nano-architectures, such as polymersomes and micelles, have attracted considerable interest for a wide range of applications, including the field of nanomedicine, due to their tunable morphology and well-defined functionality [1,2,3]. One of these architectures are bowl-shaped polymersomes, or stomatocytes, which have a unique morphology that can be exploited as drug delivery system. Besides the hydrophilic lumen and hydrophobic membrane that all types of polymersomes contain, and that allows the respective encapsulation of hydrophilic and hydrophobic cargoes, they have an additional nanocavity, which is in direct contact with the outside environment. This cavity has, in previous reports, been loaded with a large variety of cargoes, such as inorganic functional nanoparticles (i.e., Pt and MnO_2_) [4,5,6], bio-organic catalysts (i.e., enzyme molecules) [7,8], therapeutic payloads (i.e., chemotherapeutic drugs and photosensitizers) [9], and fluorescent dyes [10].

Fluorescent labeling is a standard method used for all nanoparticles to track them when they are taken up by cells or administered in vivo. Labeling is mostly performed via traditional approaches, including chemical conjugation and physical encapsulation of conventional dyes [11,12]. However, aggregation-induced quenching (ACQ) and photobleaching during the preparation and application are often hard to avoid [13,14,15]. These issues can be overcome by using aggregation-induced emission (AIE). The AIEgenic moieties attain their fluorescence in the aggregated state, and show higher resistance against photobleaching. Recently, AIE has also been introduced in polymersomes [16,17,18]. By conjugating AIE moieties to the hydrophobic domain of the block copolymers that constitute the polymersomes, the particles were endowed with intrinsic fluorescence. This feature could furthermore be combined with photo-mediated photodynamic therapy [19,20,21].

Herein, we have extended the AIEgenic features to bowl-shaped biodegradable stomatocytes (AIE stomatocytes), which were furthermore equipped with therapeutic function. The building blocks used for this design were based on our previously reported AIE incorporated amphiphilic copolymers, of which the resulting polymersomes were effectively used for both fluorescent imaging and generation of reactive oxygen species (ROS) for photodynamic therapy [22]. The additional stomatocyte nanocavity was used for the loading of enzymes (catalase and glucose oxidase), which were cross-linked after encapsulation for construction of compartmentalized cross-linked enzymatic nano-aggregates (c-CLEnA) to prevent leaching. The presence of the enzymes and the asymmetric architecture endowed the stomatocytes with motile behavior in presence of the enzyme substrates glucose or hydrogen peroxide. Autonomous motion significantly enhanced the intracellular delivery of AIE stomatocytes. Both the AIE stomatocytes and cross-linked enzyme-loaded AIE stomatocytes (AIEgenic c-CLEnA) were successfully employed for photodynamic therapy towards HeLa cells upon laser irradiation. Furthermore, the generation of H_2_O_2_ by glucose oxidase from the AIEgenic c-CLEnA, also enabled a second chemotherapeutic strategy in the absence of laser illumination.

## 2. Materials and Methods

### 2.1. Materials

Poly(ethylene glycol) methyl ether (mPEG, *M*_n_ 2 kDa) was obtained from Rapp Polymers. Tetrahydrofuran (THF) was obtained from Biosolve Chimie. XPhos Pd G2, potassium acetate (≥99%), bis(pinacolato)diboron (99%), tetrakis(triphenylphosphine)palladium(0) (99%), titanium(IV) chloride (99%), zinc powder (99%), 4-chloro-4-hydroxybenzophenone (98%), 4-bromobenzophenone (98%), trifluoromethanesulfonic acid (99%), potassium carbonate (K_2_CO_3_, 99%), cesium fluoride (CsF, 99%), 2,2-bis(hydroxymethyl)propionic acid (99%), hydrogen peroxide (H_2_O_2_), 2′,7′-dichlorofluorescin diacetate (DCFH-DA), and 3-(4,5-dimethylthiazol-2-yl)-2,5-diphenyl tetrazolium bromide (MTT) were supplied by Sigma-Aldrich (St. Louis, MI, USA). Bis(4-methoxyphenyl)methanone (98%) was from TCI. Dialysis membranes were purchased from Spectra/Pro^®^ (MWCO 12,000–14,000). Catalase from bovine liver (≥10,000 units/mg), glucose oxidase from *Aspergillus niger* (Type VII, ≥100,000 units/g), genipin (≥98% (HPLC)), Wheat Germ Agglutinin (WGA)-Alexa Fluor^TM^ 488 conjugate, Wheat Germ Agglutinin (WGA)-Alexa Fluor^TM^ 594 conjugate, Hoechst 33342, propidium iodide (PI), Gibco fetal bovine serum (no mycoplasma, FBS), Dulbecco’s Modified Eagle Medium (DMEM) with high, low, and no glucose, phosphate buffered saline (1×, pH 7.4, PBS), trypsin-EDTA, penicillin–streptomycin (5000 U/mL), and live cell imaging solution were obtained from Thermo Fisher Scientific. Amicon Ultra-0.5 Centrifugal Filter Unit (0.5 mL, 10 kDa) and Ultrafree-MC Centrifugal Filters (0.5 mL, 0.1 μm pore size) were purchased from Millipore. Ultrapure Milli-Q water (18.2 MΩ), produced by a Merck Millipore Q-Pod system with a Millipore Express 40 filter (0.22 μm), was used for all experiments. All of the chemicals were used directly without further treatment.

### 2.2. Preparation of Spherical and Shape Changed Polymersomes

Amphiphilic block-co-polymers PEG-P(AIE) were synthesized according to the previously reported methodology [22]. To prepare the AIE polymersomes, PEG_44_-P(AIE)_14_ was dissolved in THF (2 mg/mL) in a 4 mL glass vial with a magnetic stirring bar and sealed with a rubber septum. After stirring for 10 min, 0.5 mL of ultrapure Milli-Q water was added to the polymer solution through a syringe pump (Chemyx, Inc., Fusion 100, KR Analytical Limited, Stafford, TX, USA) with a speed of 0.25 mL/h. Then, the resulting cloudy solution was transferred into a pre-hydrated dialysis bag (Spectra/Pro^®^, MWCO 12,000–14,000, 2 mL/cm, Rancho Dominguez, CA, USA) for dialysis against 0 mM NaCl or 100 mM NaCl solution at 4 °C for at least 24 h. Dynamic light scattering (DLS, Nano ZSP, Malvern Instruments, Malvern, UK), scanning electron microscopy (SEM, FEI Quanta 200 3D FEG, Thermo Fisher Scientific, Waltham, MA, USA), and cryogenic transmission electron microscopy (cryo-TEM, TU/e CryoTitan, Thermo Fisher Scientific, Waltham, MA, USA) were used for size and morphological characterization. For characterization of the fluorescence, a Spark^®^ 10M microplate reader (TECAN, Männedorf, Switzerland), and two-photon/confocal laser scanning microscopy (TP-CLSM, Leica TCS SP8X, Wetzlar, Germany) were applied.

### 2.3. Preparation of Cross-Linked Enzymes-Loaded AIE Stomatocytes (AIEgenic c-CLEnA)

The procedure for the preparation of AIEgenic c-CLEnA was adapted from a previously reported protocol [8]. Briefly, AIE stomatocytes in 100 mM NaCl were concentrated to 4 mg/mL using an Amicon Ultra-0.5 Centrifugal Filter Unit (0.5 mL, 10 kDa, Merck Millipore, Darmstadt, Germany) (10,000 rpm, 5 min). Then 10 mg/mL glucose oxidase (GOx, 50 μL) in Milli-Q water, 10 mg/mL catalase (CAT, 50 μL) in Milli-Q water, and 1 mg/mL genipin (200 μL) were added to 250 μL AIE stomatocytes solution in an Eppendorf tube (1.5 mL). The mixture was kept under gentle stirring (1400 rpm) at 25 °C. After 24 h, purification was conducted using a spin filter (Merck Millipore, Darmstadt, Germany) (14,000 rpm, 4 min) and the samples were washed with Milli-Q water twice. Finally, the AIEgenic c-CLEnA were redispersed in 500-μL Milli-Q water.

### 2.4. Motion Studies by NanoSight Tracking Analysis (NTA)

The autonomous motion behaviors of AIEgenic c-CLEnA were characterized using nanoparticle tracking analysis using a NanoSight S300. AIEgenic c-CLEnA were suspended in H_2_O_2_ (0, 5, 20, 50, 100 mM), and cell culture medium DMEM without glucose, with low glucose (1 mg/mL), and high glucose (4.5 mg/mL). The approximate concentration of each sample was 10^7^ and 10^8^ particles per mL. For a typical experiment, 1 mL sample solution was loaded in the NTA sample chamber via a syringe. Then, the movement of the stomatocytes was recorded for 30 s. To ensure reproducibility, each sample was measured three times. The NTA 2.2 software was used to track the motion trajectory of single particles. Based on the extracted X and Y coordinates, the mean squared displacement (MSD) and velocity were determined according to the reported method [4,5,6,7]. For each sample, 30 nanoparticles were tracked for 30 s.

### 2.5. Cell Studies

Cell culture: HeLa cells were cultured in DMEM medium containing 10% FBS and 1% penicillin–streptomycin in the cell incubator (Thermo Fisher Scientific, Waltham, MA, USA) at 37 °C with an atmosphere of 5% CO_2_ and 70% humidity. Trypsin-EDTA was used for cell dissociation when passing and seeding the cells to cell culture flasks (Thermo Fisher Scientific, Waltham, MA, USA), 96-well-plates (Thermo Fisher Scientific, Waltham, MA, USA), or μ-Slide 8 wells (ibidi, Gräfelfing, Germany).

Cytotoxicity evaluation: MTT assay was used to evaluate the toxicity of AIE stomatocytes and AIEgenic c-CLEnA in presence of cell culture DMEM medium without glucose, with high or low glucose. HeLa cells were seeded into 96-well-plates (2 × 10^3^ cells per well) and incubated with the stomatocyte samples for 4 and 24 h. Subsequently, the cells were washed three times with PBS, and fresh cell culture medium (without/with low/with high glucose) containing MTT reagent (100 μL per well) was added. After 4 h incubation, the medium was removed, followed by the addition of dimethyl sulfoxide (100 μL) to dissolve the formazan crystals. Thereafter, the 96-well plates were gently shaken for 5 min and the absorbance at 490 nm was measured using a micro-plate reader (TECAN Spark^®^, Männedorf, Switzerland).

Internalization of AIE-polymersomes in cells: HeLa cells were seeded in a μ-Slide 8 well containing DMEM medium with 10% FBS and 1% penicillin–streptomycin. After incubation with AIE stomatocytes for 24 h, the cell culture medium was refreshed with live cell imaging solution. For the AIEgenic c-CLEnA, the incubation time was 4 h. Thereafter, the cell membrane was stained with wheat germ agglutinin (WGA)-Alexa Fluor^TM^ (Thermo Fisher Scientific, Waltham, MA, USA ) 488 conjugate (WGA-AF488) for 10 min. Fluorescence images were acquired using TP-CLSM (Leica TCS SP8X, Wetzlar, Germany) equipped with a microscope incubator (Okolab, Pozzuoli, Italy) for live cell imaging.

Intracellular generation of reactive oxygen species (ROS): HeLa cells were incubated in ibidi-8 wells for 24 h, followed by adding AIE stomatocytes (100 μg/mL) in DMEM medium containing 10% FBS and 1% penicillin–streptomycin. After incubation for 24 h, HeLa cells were washed with live cell imaging solution for three times to remove the free AIE stomatocytes. Thereafter, the cell membrane and nucleus were stained with wheat germ agglutinin (WGA)-Alexa Fluor^TM^ 594 conjugate (WGA-AF594) and Hoechst 33342 for 10 min respectively. The ROS was detected by staining the cells with the fluorescent probe DCFH-DA for 30 min. TP-CLSM (Leica TCS SP8X) equipped with a microscope incubator (Okolab, Pozzuoli, Italy) was then used for characterization of ROS generation.

Cell apoptosis induced by AIE stomatocytes: HeLa cells were seeded and cultured in DMEM medium containing 10% FBS and 1% penicillin–streptomycin in ibidi-8 wells for 24 h. Before incubation with AIE stomatocytes, the cell culture medium was refreshed. HeLa cells were incubated with AIE stomatocytes (100 μg/mL) in DMEM medium for another 24 h. Then the cells were washed with PBS, followed by staining the cell membrane and nucleus with WGA-AF488 and Hoechst 33342 for 10 min. To observe the cell apoptosis in situ, PI was added to the live cell imaging solution before capturing the fluorescence images using TP-CLSM with/without laser irradiation (TP-NIR wavelength was set at 760 nm, and the output laser power was 0.73 W).

Active intracellular transportation: After culturing HeLa cells in a μ-Slide 8 well, AIEgenic c-CLEnA (100 μg/mL) were added and incubated for 4 h. Then, PBS was used to wash the cells. The cell membrane was stained with WGA-AF488 for 10 min in live cell imaging solution. TP-CLSM was conducted to evaluate the internalization of AIEgenic c-CLEnA in the presence of DMEM medium with high or low glucose, and without glucose.

Cell apoptosis induced by AIEgenic c-CLEnA: HeLa cells were incubated in ibidi-8 wells for 24 h. AIEgenic c-CLEnA (100 μg/mL) dispersed in DMEM with high, low, and no glucose were added to HeLa cells for 4 h incubation. Subsequently, the cells were washed with PBS buffer. Fluorescence was observed using TP-CLSM, the cell membrane and nucleus were stained with WGA-AF488 and Hoechst 33342 for 10 min. PI was added to the imaging buffer as well to detect apoptotic cells. To generate ROS, a laser (760 nm) was used to illuminate the HeLa cells during TP-CLSM characterization. Cell apoptosis induced by the generation of H_2_O_2_ via the enzymatic conversion of glucose, by glucose oxidase, was conducted by incubation of HeLa cells with AIEgenic c-CLEnA (100 μg/mL) for 24 h. After refreshing the cell culture medium, fluorescent dyes WGA-AF488, and Hoechst were used to stain the cell membrane and cell nucleus. To observe the cell viability in situ, PI was added to the imaging buffer before TP-CLSM characterization.

## 3. Results and Discussion

### 3.1. Characterization of Spherical and Shape Changed AIE Polymersomes

**Figure 1:**
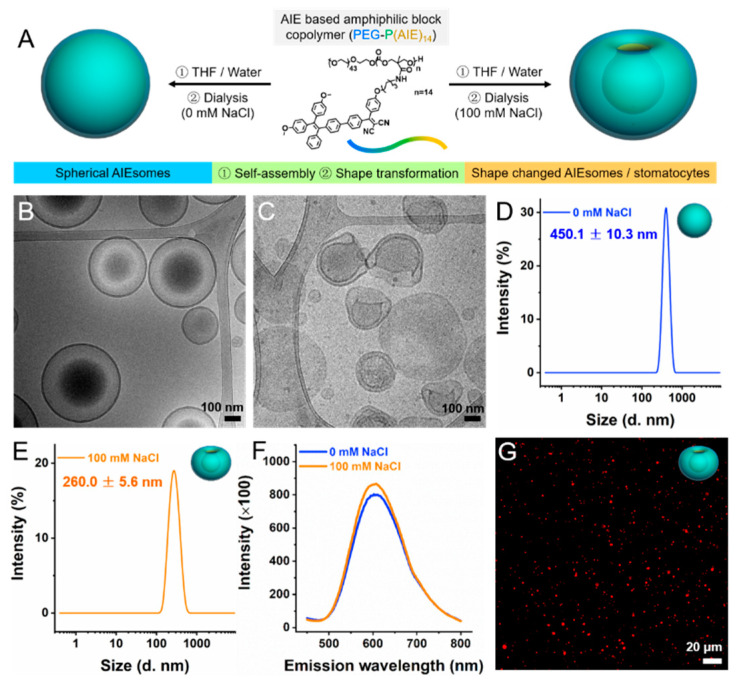
Preparation and characterization of fluorescent AIE polymersomes and stomatocytes. (**A**) Schematic depiction of their fabrication. (**B**) Cryo-TEM image of spherical AIE polymersomes. Scale bar: 100 nm. (**C**) Cryo-TEM image of AIE stomatocytes. Scale bar: 100 nm (**D**) DLS measurement of the size distribution of spherical AIE polymersomes. (**E**) DLS measurement of the size distribution after shape transformation into stomatocytes. (**F**) Fluorescence emission curves of spherical AIE polymersomes and stomatocytes (λ_ex_ = 370 nm/λ_em_ = 610 nm). (**G**) Fluorescence characterization of AIE stomatocytes using CLSM (λ_ex_ = 405 nm/λ_em_ = 600–650 nm). Scale bar: 20 μm.

AIE-based amphiphilic block copolymers were synthesized according to a previously reported method [22]. Here, poly(ethylene glycol)-block-poly(trimethylene carbonate) (PEG-TMC) was used as the structural basis. The TMC monomers were equipped with a reactive pentafluorophenyl ester substituent, which could be conveniently substituted with the AIEgenic moieties (PEG_44_-P(AIE)_14_). The AIE polymersomes were formed via the solvent switch methodology; the block copolymers were dissolved in THF (2 mg/mL), followed by controlled addition of Milli-Q water (50% vol.) using a syringe pump. Spherical AIE polymersomes were prepared by dialyzing the obtained polymer solution against 0 mM NaCl at 4 °C for 24 h to remove the organic solvent, while bowl-shaped AIE stomatocytes were dialyzed against 100 mM NaCl solution at 4 °C, as shown in Figure 1A. Morphological characterization of the as-prepared spherical and shape changed AIE polymersomes was performed using cryogenic transmission electron microscopy (cryo-TEM, Figure 1B,C) and scanning electron microscopy (SEM, Figure S1A,B). Dynamic light scattering (DLS) confirmed the size of the spherical AIE polymersomes (450.1 ± 10.3 nm) and bowl-shaped AIE polymersomes (260.0 ± 5.6 nm) with low polydispersity (PDI ≤ 0.1) (Figure 1D,E). The inherent fluorescence of the AIE polymersomes was characterized via a microplate reader and confocal laser scanning microscopy. Both of the spherical AIE polymersomes and bowl-shaped AIE polymersomes (AIE stomatocytes) exhibited strong emission as shown in Figure 1F,G, and Figure S1 (λ_ex_ = 370 nm and λ_em_ = 610 nm).

### 3.2. Enzyme Encapsulation and Autonomous Motion

**Figure 2:**
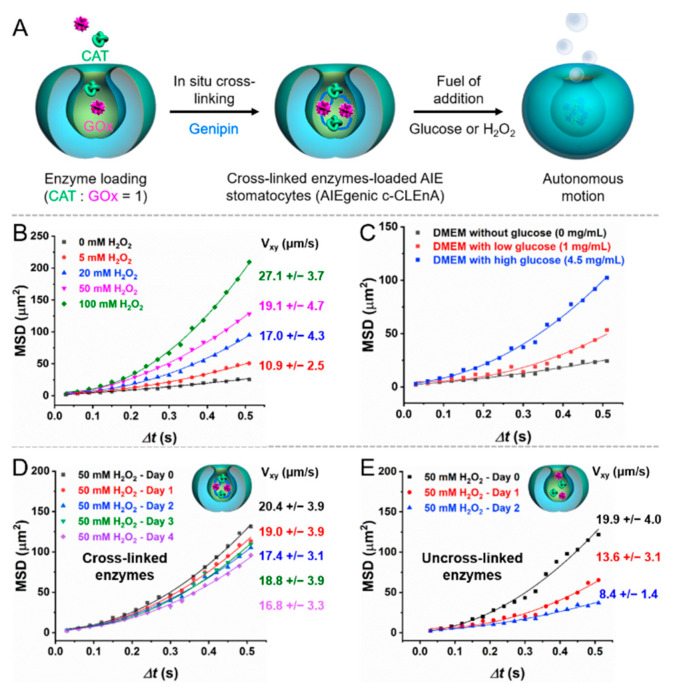
The effect of enzyme cross-linking on motility of AIE stomatocytes. (**A**) Schematic illustration of the study of motile behavior of AIEgenic c-CLEnA. Two enzymes, namely catalase (CAT) and glucose oxidase (GOx) were encapsulated and cross-linked in the lumen of stomatocytes. With the addition of fuel (i.e., glucose solution or hydrogen peroxide), AIE stomatocytes transformed into self-propelled nanomotors and exhibited autonomous motion. (**B**) MSD and velocity of AIEgenic c-CLEnA as a function of a range of H_2_O_2_ concentrations (0, 5, 20, 50, and 100 mM). Velocities were extracted from the fitting of the average MSD and theoretically calculated from MSD = (4D)*Δt* + (V^2^)(*Δt*^2^). (**C**) Autonomous movement of AIEgenic c-CLEnA analyzed by MSDs in the presence of cell culture medium DMEM with different glucose concentrations (0, 1, 4.5 mg/mL). (**D**) MSDs of AIEgenic c-CLEnA in presence of 50 mM H_2_O_2_ as a function of time. (**E**) MSDs of uncross-linked enzymes-loaded AIE stomatocytes in presence of 50 mM H_2_O_2_ as a function of time.

Compared to spherical polymersomes, bowl-shaped stomatocytes provide an extra compartment, namely a nanocavity, which can be used to encapsulate hydrophilic cargoes for various applications. To evaluate the encapsulation capacity of the as-prepared AIE stomatocytes, we subsequently selected the well-studied two enzyme cascade system containing catalase (CAT) and glucose oxidase (GOx) as cargo, as shown in Figure 2A. We furthermore applied an enzyme cross-linking method for the entrapment of the enzymes, as we previously demonstrated this to be an efficient method for increasing the enzyme local concentration in the lumen and for preventing enzyme leakage [8]. Consequently, the mild cross-linker genipin was added after enzyme encapsulation.

In the presence of hydrogen peroxide (H_2_O_2_), CAT is able to decompose hydrogen peroxide into water and oxygen, a process, which is commonly used for designing chemoenzymatic nanomotors. Glucose was able to provide a driving force as well. GOx converted glucose into gluconic acid and H_2_O_2_, which in turn was converted by CAT to provide oxygen for propulsion. The stomatocyte motility was investigated as a function of H_2_O_2_ concentration to demonstrate the encapsulation of active CAT. Motility was investigated using nanoparticle-tracking analysis (NTA) via tracking single particle motion trajectories. As shown in Figure 2B, the mean square displacements (MSDs) and velocities of the AIEgenic c-CLEnA were proportional to the H_2_O_2_ concentration, which reflects that active CAT loading was achieved successfully in the nanocavity. GOx catalyses the oxidation of glucose to gluconic acid and H_2_O_2_, and the latter product could be used as the substrate for CAT. Consequently, the loading of active GOx was confirmed by investigating the motion behavior in presence of glucose as well. Cell culture medium DMEM with different concentrations of glucose was used to record the motion of AIEgenic c-CLEnA. The highest motility was observed in medium with the highest glucose concentration (4.5 mg/mL), while approximately Brownian motion was detected in the group without glucose (Figure 2C). As demonstrated earlier, the cross-linking method efficiently prevented the leakage of the encapsulated enzymes [23,24,25]. To evaluate the stability enhancement for our AIEgenic c-CLEnA, their motile behavior as a function of time was investigated in the presence of 50 mM H_2_O_2_, as shown in Figure 2D. The MSD curves did not change significantly, and the velocity was slightly reduced from 20.4 ± 3.9 μm/s to 16.8 ± 3.3 μm/s after observation for 4 days. In contrast, uncross-linked AIE stomatocytes exhibited nearly Brownian motion at day 2, and a significant reduction in velocity from 19.9 ± 4.0 μm/s to 8.4 ± 1.4 μm/s. This noteworthy difference between uncross-linked and cross-linked AIE stomatocytes could be attributed to the re-diffusion of enzymes into the buffer after encapsulation as a function of time. Upon cross-linking, the enzymes were entrapped in the nanocavity of stomatocytes in the form of aggregates and could not leach out.

### 3.3. Intracellular Localization of AIE Stomatocytes

**Figure 3:**
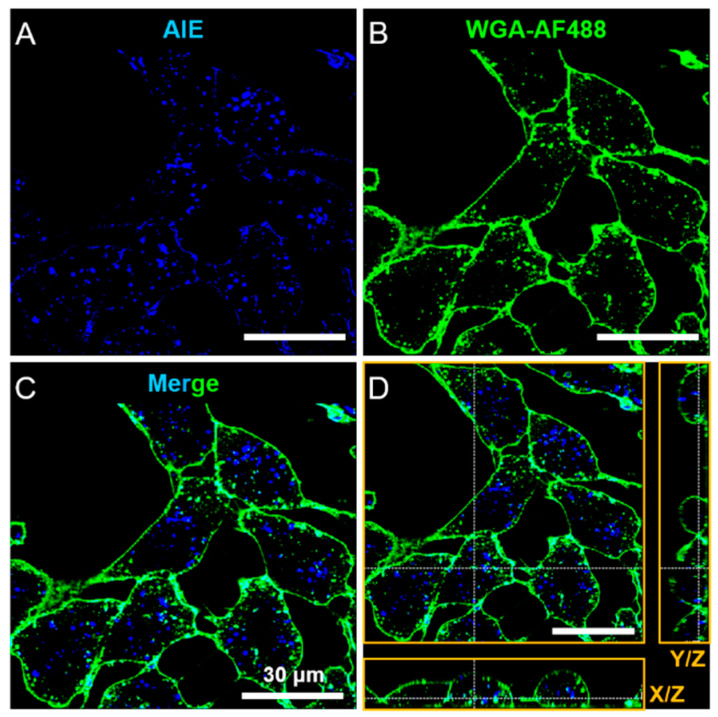
Intracellular localization of AIE stomatocytes after incubation with HeLa cells for 24 h. (**A**) CLSM image of AIE stomatocytes. (**B**) Cell membrane stained with WGA-AF488, exhibiting green fluorescence. (**C**) Co-localization assessment of HeLa cells and AIE stomatocytes by merging the two channels. Blue fluorescence originates from AIE stomatocytes, and the cell membrane exhibits a green color. (**D**) Representative orthogonal z-stack view to show the location of AIE stomatocytes inside the HeLa cells. Scale bar: 30 μm.

Before testing their therapeutic effect, the biocompatibility of the AIE stomatocytes was assessed first. We evaluated the cytotoxicity of AIE stomatocytes using the MTT assay, as shown in Figure S2. AIE stomatocytes in a range of concentrations (0, 6.25, 12.5, 25, 50, 100 μg/mL) were tested after incubation with HeLa cells for 24 h. The results demonstrated that the cell viability at all concentrations tested was higher than 90%. Secondly, particle uptake was studied. The AIE properties allowed the efficient visualization of the AIE stomatocytes using CLSM and the HeLa cell membrane was stained with WGA-AF488 (Figure 3A–C). To make sure the AIE stomatocytes were localized in the cytoplasm, instead of attached on the surface of the cells, z-stack imaging was performed. A representative orthogonal z-stack view demonstrated the AIE stomatocytes were intracellularly localized, as shown in Figure 3D.

### 3.4. Photo-Mediated Therapy of AIE Stomatocytes toward HeLa Cells

Besides providing intrinsic fluorescence, the AIEgenic segments also endowed the AIE stomatocytes with photodynamic therapeutic function by producing reactive oxygen species (ROS) upon irradiation [19,22]. Consequently, there was no necessity to encapsulate a photosensitizer to achieve photo-mediated anticancer therapy. A series of in vitro experiments were designed and conducted to assess the therapeutic performance of AIE stomatocytes. The fluorescent signal of the AIE stomatocytes was detected using the same channel as for the nucleus-staining agent Hoechst. To clearly distinguish the particles from the nucleus, a z-stack image was obtained. As demonstrated in Figure S3, the AIE stomatocytes were localized in the cytoplasm. Thereafter, we evaluated the ROS generation within HeLa cells using CLSM by detecting the fluorescence of the ROS indicator, DCFH-DA, as shown in Figure S4.

**Figure 4:**
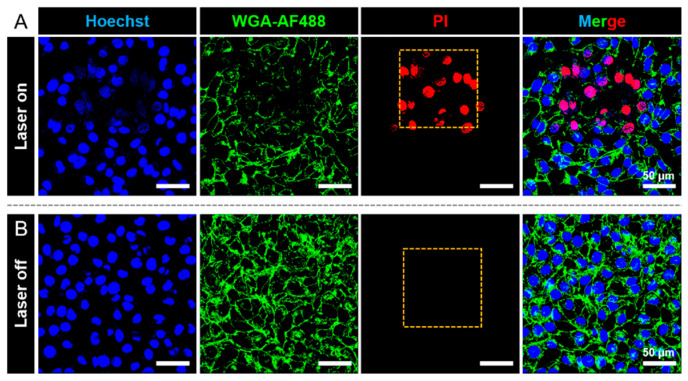
Phototherapeutic performance of AIE stomatocytes toward HeLa cells. (**A**) CLSM images of HeLa cells after PDT treatment. The laser selectively irradiated a specific area to induce the generation of toxic ROS. The corresponding irradiated region is indicated in the PI with a yellow square. Cell apoptosis was only detected in the laser-irradiated area, as apoptotic cells were marked by PI. (**B**) CLSM images of HeLa cells with AIE stomatocytes in the absence of laser irradiation. The nucleus was stained with Hoechst (blue signal), and the cell membrane was stained with WGA-AF488 (green signal). Apoptotic cells stained with PI were not detected in the red channel (PI channel). Scale bar: 50 μm.

Next, cell apoptosis after PDT treatment was studied with propidium iodide (PI) dead cell staining. As shown in Figure 4, only the irradiated region displayed a PI signal, which shows the spatial control of the PDT treatment. In the absence of laser irradiation, HeLa cells in the presence of AIE stomatocytes were still alive since no PI signal could be detected. With increased irradiation time (approximately 3 min in total), all of the HeLa cells that were exposed to the laser were killed, while HeLa cells outside the exposed region were still in good condition, as observed in Figure S5. Furthermore, as a control group HeLa cells were irradiated with laser light in the absence of AIE stomatocytes under the same experimental conditions (760 nm). As shown in Figure S6, no dead cells could be observed with PI staining, which means the incident laser power was safe to the HeLa cells. Consequently, cell apoptosis in the presence of AIE stomatocytes was mainly induced by photo-mediated therapy, instead of photo-toxicity.

### 3.5. Active Intracellular Delivery via AIEgenic c-CLEnA

**Figure 5:**
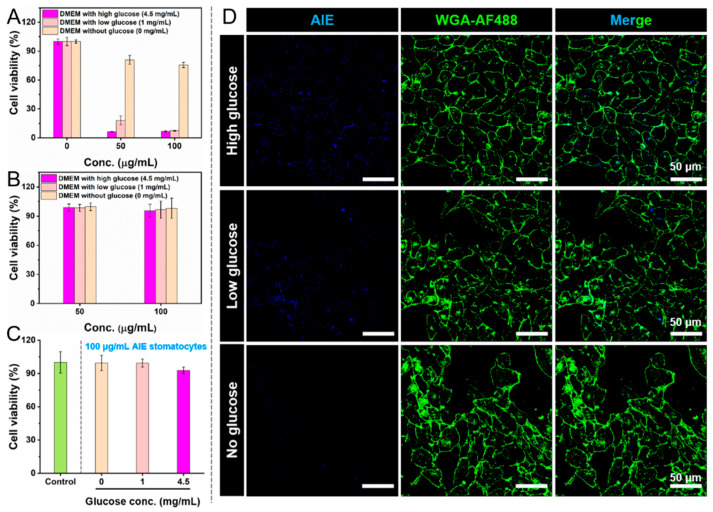
Cytotoxicity and cellular uptake of AIEgenic c-CLEnA toward HeLa cells. (**A**) Cell viability after 24 h incubation in the presence of cell culture medium with high (4.5 mg/mL), low (1 mg/mL), and no (0 mg/mL) glucose, as determined by MTT assay. (**B**) Cell viability after 4 h incubation with AIEgenic c-CLEnA. (**C**) Cell viability of AIE stomatocytes without cross-linking enzymes incubated in cell culture medium with different glucose concentrations (0, 1, 4.5 mg/mL) for 4 h. (**D**) CLSM fluorescent images of HeLa cells after 4 h incubation with AIEgenic c-CLEnA in cell culture medium with high, low, and no glucose. Blue fluorescence represents the stomatocyte samples, and the green signal originates from the cell membrane staining with WGA-AF488. Scale bar: 50 μm.

After evaluation of the therapeutic effect of AIE stomatocytes, the enzyme-loaded variants were tested as well. Regular cell culture medium DMEM contains glucose, which could be used as fuel to propel the AIEgenic c-CLEnA. It has been demonstrated that a drug delivery system with motile properties can enhance intracellular delivery, by improving the interaction with the cells and by facilitating translocation across the cell membrane [26,27,28,29,30]. Furthermore, the generation of the intermediate H_2_O_2_ produced by GOx could also be used as a therapeutic agent to treat cancer cells. Before evaluating the AIEgenic c-CLEnA in active intracellular delivery and therapeutic performance, we first tested their cytotoxicity towards HeLa cells in the presence of DMEM with high, low, and no glucose. As shown in Figure 5A, lower cell viability was detected in both the low and high glucose medium after 24 h incubation with AIEgenic c-CLEnA (50 and 100 μg/mL). More than approximately 80% of HeLa cells were still alive when exposed to the cell culture medium DMEM without glucose, which demonstrated that the cell-killing capacity was caused by H_2_O_2_ generation. For the remainder of the experiments the incubation time was restricted to 4 h, to assess adequately the effect of the other therapeutic modalities. It is worth to mention that cell viability was higher than 90% among all of the groups after 4 h incubation, as confirmed in Figure 5B. As a control group, empty AIE stomatocytes (without cross-linked enzymes, 100 μg/mL) were tested in the corresponding DMEM buffers (Figure 5C). The applied conditions, including incubation time (4 h) and buffer (high, low, and no glucose), were all compatible with the HeLa cells as cell viabilities were all up to 90%. To demonstrate that cellular uptake could be enhanced using AIEgenic c-CLEnA, fluorescent images of HeLa cells after 4 h incubation in the presence of DMEM with high, low, and no glucose were compared using CLSM (Figure 5D). Additionally, quantitative analysis of the corresponding images in Figure 5D (AIE channel) was conducted by ImageJ, as shown in Figure S7. Fluorescent signals of AIEgenic c-CLEnA were only observed when medium with glucose was applied, and the best performance in cellular uptake occurred in the presence of medium with high glucose.

### 3.6. H_2_O_2_-Mediated/Photo-Mediated Therapy Using AIEgenic c-CLEnA

**Figure 6:**
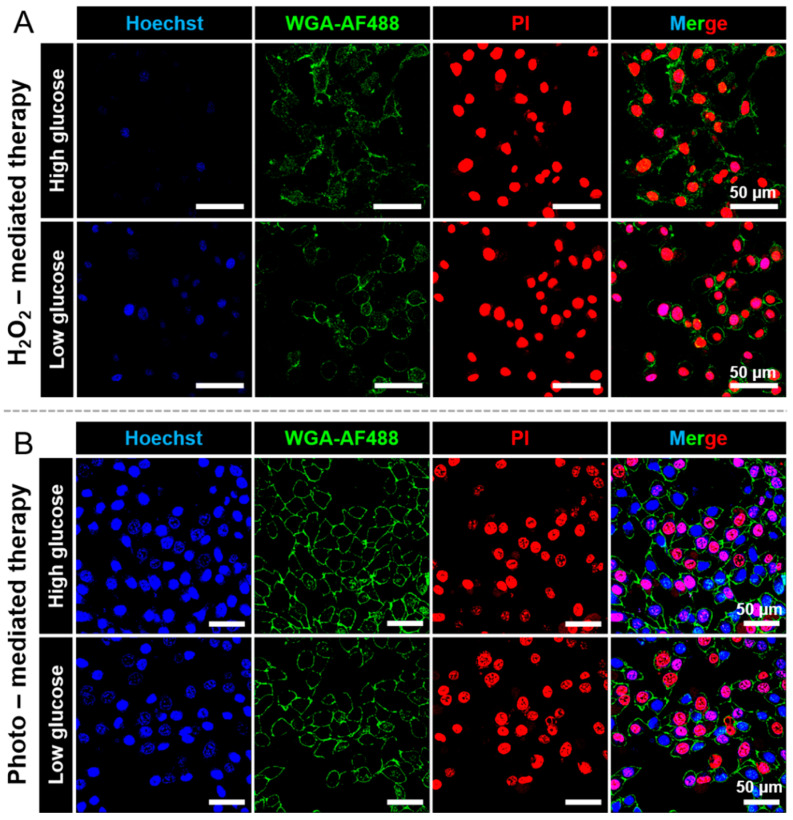
Therapeutic evaluation of AIEgenic c-CLEnA via H_2_O_2_-mediated and photo-mediated cell killing. (**A**) CLSM images of HeLa cells after incubation with AIEgenic c-CLEnA for 24 h. PI was used to indicate the cell viability after H_2_O_2_-mediated cell killing. (**B**) CLSM images of HeLa cells after PDT treatment activated by laser irradiation (photo-mediated cell killing). Scale bar: 50 μm.

Having validated the PDT therapeutic effect of AIE stomatocytes (without cross-linked enzymes), and the H_2_O_2_-mediated cell killing of AIEgenic c-CLEnA, the latter particles were used for a combination approach, including H_2_O_2_-mediated and photo-mediated cell killing (PDT). To confirm the therapeutic performance, HeLa cells were treated with AIEgenic c-CLEnA for 24 h, followed by washing with buffer (live cell imaging solution) to remove free nanoparticles. The cellular nucleus and membrane were stained with Hoechst and WGA-AF488 to allow visualization by fluorescence. Apoptotic cells after treatment were monitored by adding PI to the imaging buffer. As shown in Figure 6A, cell apoptosis was observed in the group of cell culture medium with high glucose (4.5 mg/mL) and low glucose (1 mg/mL).

The unique property of AIE stomatocytes, namely photo-mediated PDT, provided a second approach to achieve anticancer therapy. As demonstrated in Figure 5, enhanced intracellular delivery could be obtained after incubation with HeLa cells for 4 h. Therefore, to evaluate the therapeutic effect of photo-mediated treatment, HeLa cells were first incubated with AIEgenic c-CLEnA for 4 h, after which Hoechst and WGA-AF488, alongside PI were added before microscopic observation. After laser irradiation, for both the low and high glucose conditions, cell apoptosis could be detected, as shown in Figure 6B. While in the control groups, HeLa cells with AIEgenic c-CLEnA in the absence of laser irradiation and HeLa cells in the absence of AIEgenic c-CLEnA with laser irradiation were still in a good condition (Figures S8 and S9). In addition, HeLa cells were treated with laser irradiation after incubation with AIEgenic c-CLEnA, in cell culture medium without glucose as another control group. After photo-mediated treatment, the HeLa cells were still intact since no apoptotic cells could be observed in the PI channel (Figure S10). The noteworthy difference in the phototherapeutic effect between the HeLa cells in DMEM without glucose and the ones with low/high glucose could be attributed to the enhanced intracellular uptake by the motile properties in the presence of glucose, which has been confirmed in Figure 5. The abovementioned results show that AIEgenic c-CLEnA can achieve therapeutic efficacy via two ways, namely H_2_O_2_-mediated and photo-mediated PDT cell killing. The incubation time for cellular uptake was significantly shortened due to the motile properties of AIEgenic c-CLEnA. The therapeutic performance of AIEgenic c-CLEnA can thus be manipulated by selecting the cell culture medium (with or without glucose) and incubation time (4 or 24 h).

## 4. Conclusions

In summary, AIE stomatocytes with inherent fluorescent and photodynamic therapeutic properties were constructed. The bowl-shaped AIE stomatocyte morphology was attained by controlling the dialysis conditions. The AIEgens formed an integral part of the polymer building blocks of which the stomatocytes were assembled, which endowed the particles with intrinsic fluorescence and ROS generation capacity. The loading of the stomatocyte nanocavity with the cross-linked enzymes glucose oxidase and catalase provided the particles with motile behavior upon conversion of glucose and/or H_2_O_2_. This specific feature resulted in enhanced intracellular delivery of the nanoparticles. Furthermore, two types of therapy could be executed. Using both AIE stomatocytes (without enzymes) and cross-linked enzymes-loaded AIE stomatocytes (AIEgenic c-CLEnA) with laser irradiation led to efficient cell killing via photo-mediated PDT. Moreover, AIEgenic c-CLEnA also allowed cell killing via the production of H_2_O_2_, meanwhile enhancing the intracellular delivery. AIEgenic c-CLEnA therefore prove to be a multifunctional therapeutic platform, where the different features act synergistically in cancer cell killing.

## Data Availability

The data presented in this work are available upon request from the corresponding authors.

## Supplementary Materials

The following are available online at https://www.mdpi.com/article/10.3390/pharmaceutics13111833/s1, Figure S1: (A) Morphological characterization of spherical AIE polymersomes using SEM. Scale bar: 1 μm. (B) SEM images of bowl-shaped AIE stomatocytes. Scale bar: 1 μm. (C) Fluorescent characterization of spherical AIE poly-mersomes using CLSM (λex = 405 nm/λem = 600–650 nm). Scale bar: 20 μm, Figure S2: Cell viability of HeLa cells after 24 h incubation with AIE stomatocytes, evaluated by the MTT assay, Figure S3: Intracellular localization of AIE stomatocytes determined by CLSM. Blue signals originated from the cell nucleus and AIE stomatocytes. The cell membrane was stained with WGA-AF488 (green fluorescence). Representative orthogonal z-stack view confirms that the AIE stomatocytes were in the cytoplasm. Scale bar: 30 μm, Figure S4: CLSM images of reactive oxygen species (ROS) generation within HeLa cells after incubation with AIE stomatocytes for 24 h. Red fluorescence indicates the cell membranes which were stained with WGA-AF594. Cell nucleus were stained with Hoechst 33342 which exhibit blue fluorescence, The green signal was from the ROS indicator, DCFH-DA. Scale bar: 50 μm, Figure S5: Phototherapeutic effect of AIE stomatocytes in HeLa cells as determined by TP-CLSM. (A) ROI region irradiated with NIR laser. Apoptotic cells stained with PI were only detected in the irradiated area. (B) ROI area in the absence of NIR laser irradiation. Scale bar: 50 μm, Figure S6: Cell viability in the absence of AIE stomatocytes under laser irradiation, as evaluated using CLSM. PI was added to the live cell imaging buffer to monitor the dead cells. No obvious cell apoptosis was detected. Scale bar: 50 μm, Figure S7: Quantitative analysis the CLSM images of Figure 5D (AIE channel) by ImageJ, Figure S8: CLSM images of HeLa cells with AIEgenic c-CLEnA (incubation time was 4 h) in the absence of laser irradiation. Scale bar: 50 μm, Figure S9: CLSM images of HeLa cells after laser irradiation (ca. 6 min). Scale bar: 50 μm, Figure S10: CLSM images of HeLa cells after incubation for 4 h with AIEgenic c-CLEnA in cell culture medium without glucose, followed by photo-mediated treatment. Scale bar: 50 μm.
