# Supplementary material for: Therapeutic Stomatocytes with Aggregation Induced Emission for Intracellular Delivery
Source: Pharmaceutics. 2021 Nov 2;13(11):1833. doi: 10.3390/pharmaceutics13111833 (PMC8617661; doi:10.3390/pharmaceutics13111833)
Supplement: Supplementary file 1 [file pharmaceutics-13-01833-s001.zip › pharmaceutics-1420465-supplementary.pdf]

pharmaceutics

Supplementary Materials: Therapeutic Stomatocytes with Aggregation Induced Emission for Intracellular Delivery

Jingxin Shao, Shoupeng Cao, Hanglong Wu, Loai K. E. A. Abdelmohsen and Jan C. M. van Hest

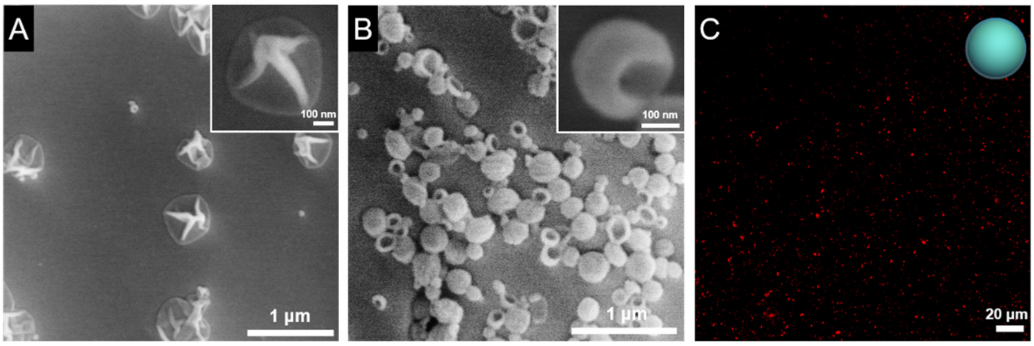

Figure S1. (A) Morphological characterization of spherical AIE polymersomes using SEM. Scale bar: 1 µm. (B) SEM images of bowl-shaped AIE stomatocytes. Scale bar: 1 µm. (C) Fluorescent characterization of spherical AIE polymersomes using CLSM (λex = 405 nm / λem = 600-650 nm). Scale bar: 20 µm.

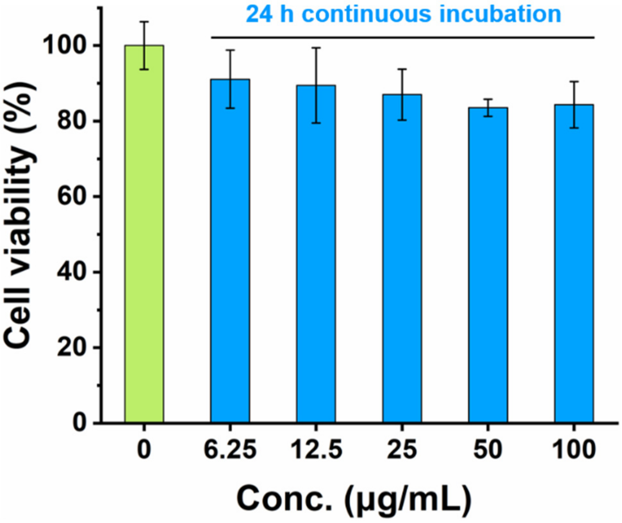

Figure S2. Cell viability of HeLa cells after 24 h incubation with AIE stomatocytes, evaluated by the MTT assay.

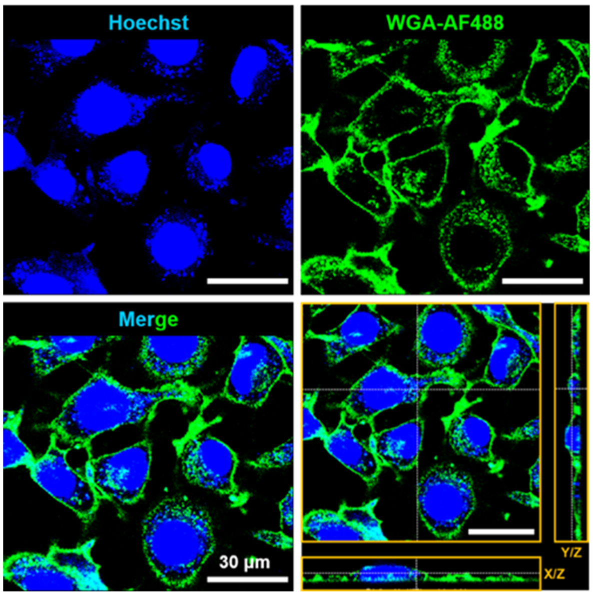

Figure S3. Intracellular localization of AIE stomatocytes determined by CLSM. Blue signals originated from the cell nucleus and AIE stomatocytes. The cell membrane was stained with WGA-AF488 (green fluorescence). Representative orthogonal z-stack view confirms that the AIE stomatocytes were in the cytoplasm. Scale bar: 30 μm.

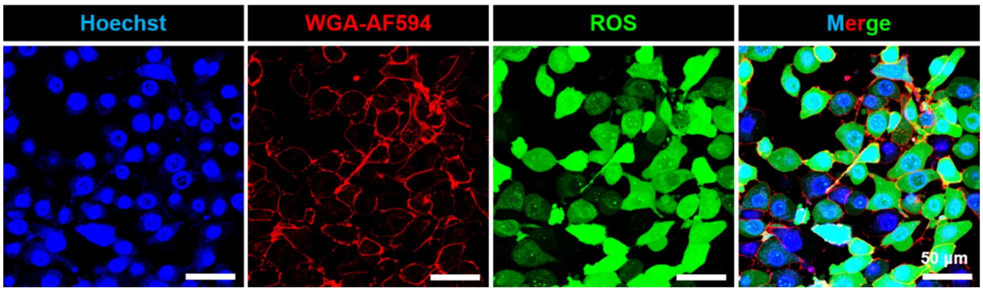

Figure S4. CLSM images of reactive oxygen species (ROS) generation within HeLa cells after incubation with AIE stomatocytes for 24 h. Red fluorescence indicates the cell membranes which were stained with WGA-AF594. Cell nucleus were stained with Hoechst 33342 which exhibit blue fluorescence, The green signal was from the ROS indicator, DCFH-DA. Scale bar: 50 μm.

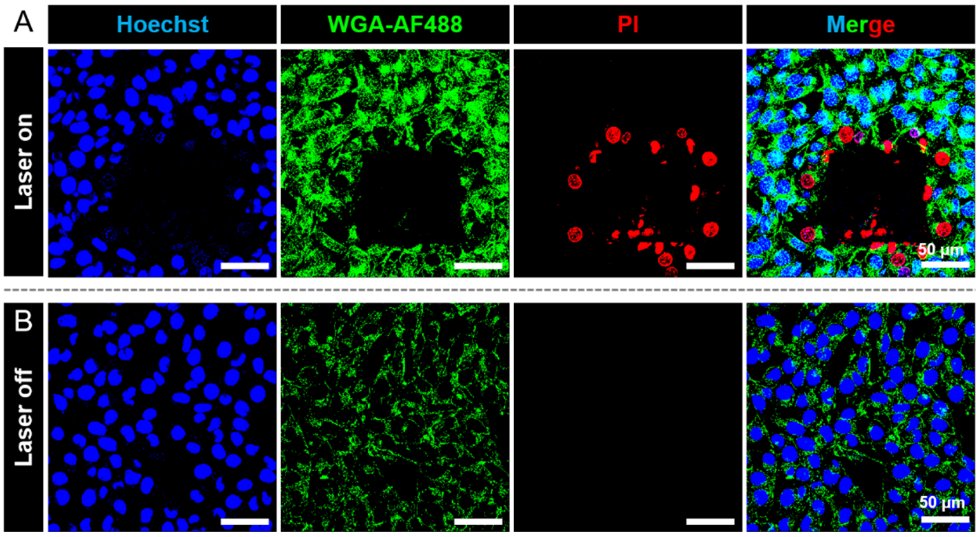

Figure S5. Phototherapeutic effect of AIE stomatocytes in HeLa cells as determined by TP-CLSM. (A) ROI region irradiated with NIR laser. Apoptotic cells stained with PI were only detected in the irradiated area. (B) ROI area in the absence of NIR laser irradiation. Scale bar: 50 μm.

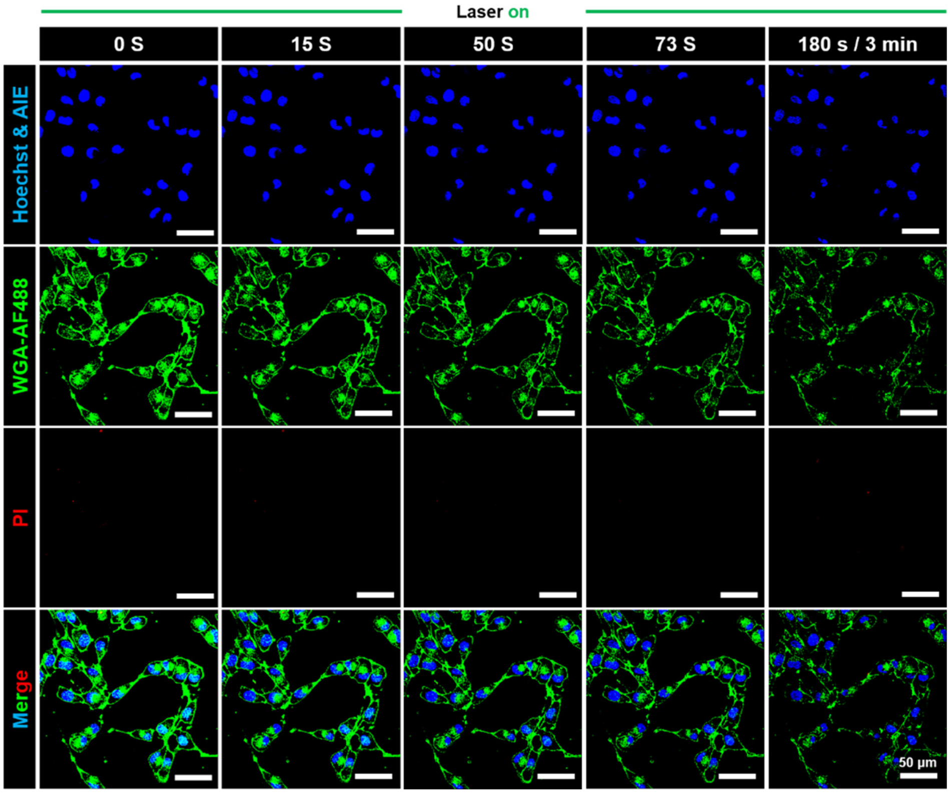

Figure S6. Cell viability in the absence of AIE stomatocytes under laser irradiation, as evaluated using CLSM. PI was added to the live cell imaging buffer to monitor the dead cells. No obvious cell apoptosis was detected. Scale bar: 50 μm.

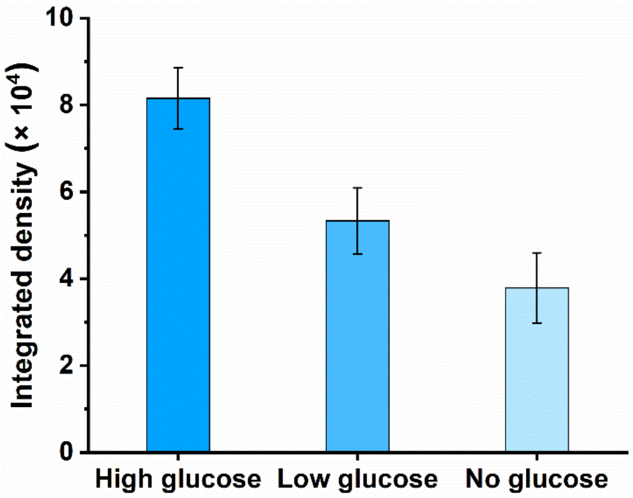

Figure S7. Quantitative analysis the CLSM images of Figure 5D (AIE channel) by ImageJ.

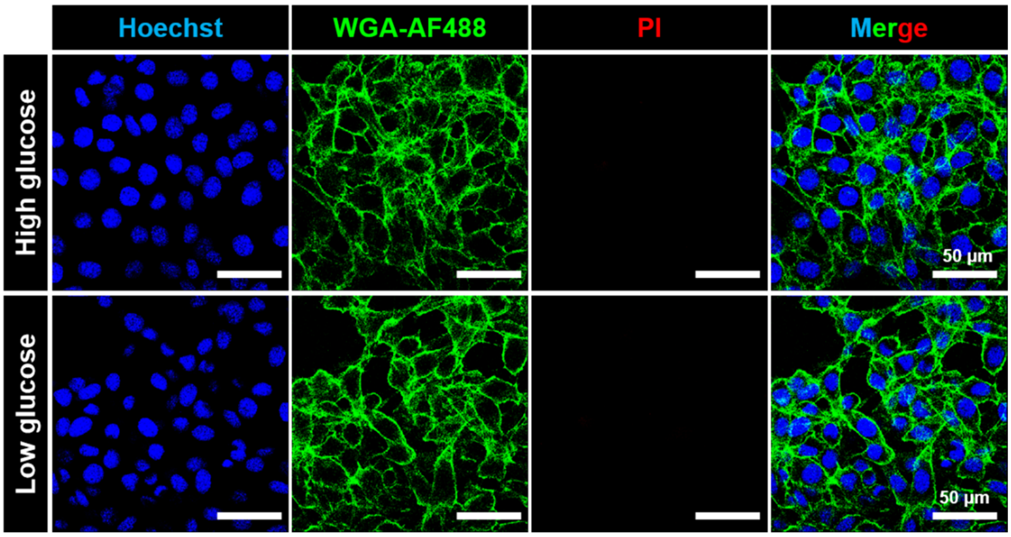

Figure S8. CLSM images of HeLa cells with AIEgenic c-CLEnA (incubation time was 4 h) in the absence of laser irradiation. Scale bar: 50 μm.

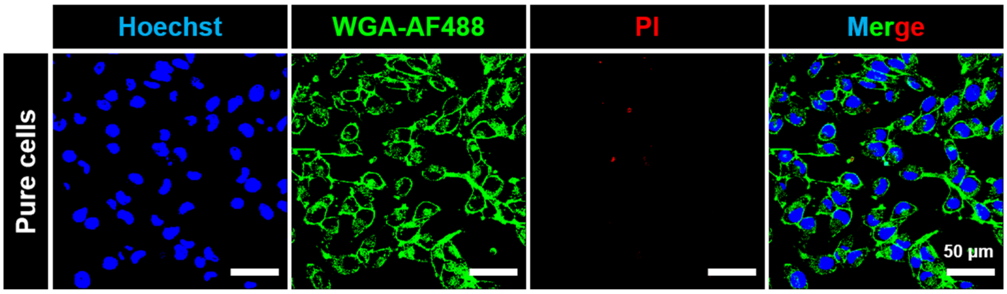

Figure S9. CLSM images of HeLa cells after laser irradiation (ca. 6 min). Scale bar: 50 μm.

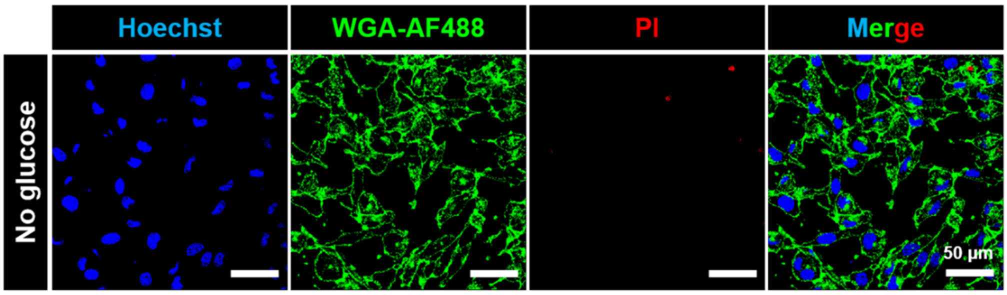

Figure S10. CLSM images of HeLa cells after incubation for 4 h with AIEgenic c-CLEnA in cell culture medium without glucose, followed by photo-mediated treatment. Scale bar: 50 μm.
